# MRI-traceable theranostic nanoparticles for targeted cancer treatment

**DOI:** 10.7150/thno.48811

**Published:** 2021-01-01

**Authors:** Tareq Anani, Shiva Rahmati, Nayer Sultana, Allan E. David

**Affiliations:** Department of Chemical Engineering, Samuel Ginn College of Engineering, Auburn University, Auburn, AL 36849, USA

**Keywords:** MRI-guided therapy, combination therapy, multimodal imaging, active targeting, magnetic targeting

## Abstract

Current cancer therapies, including chemotherapy and radiotherapy, are imprecise, non-specific, and are often administered at high dosages - resulting in side effects that severely impact the patient's overall well-being. A variety of multifunctional, cancer-targeted nanotheranostic systems that integrate therapy, imaging, and tumor targeting functionalities in a single platform have been developed to overcome the shortcomings of traditional drugs. Among the imaging modalities used, magnetic resonance imaging (MRI) provides high resolution imaging of structures deep within the body and, in combination with other imaging modalities, provides complementary diagnostic information for more accurate identification of tumor characteristics and precise guidance of anti-cancer therapy. This review article presents a comprehensive assessment of nanotheranostic systems that combine MRI-based imaging (T1 MRI, T2 MRI, and multimodal imaging) with therapy (chemo-, thermal-, gene- and combination therapy), connecting a range of topics including hybrid treatment options (*e.g.* combined chemo-gene therapy), unique MRI-based imaging (*e.g.* combined T1-T2 imaging, triple and quadruple multimodal imaging), novel targeting strategies (*e.g.* dual magnetic-active targeting and nanoparticles carrying multiple ligands), and tumor microenvironment-responsive drug release (*e.g.* redox and pH-responsive nanomaterials). With a special focus on systems that have been tested *in vivo*, this review is an essential summary of the most advanced developments in this rapidly evolving field.

## Introduction

Cancer is a leading cause of mortality, with 606,520 cancer-related deaths projected to occur in the United States in 2020 1. Despite recent advances in the fields of cancer diagnosis and therapy, the path to curative therapies remains littered with significant hurdles that must be overcome. Current treatment options, including surgery, radiotherapy, and chemotherapy, are invasive, painful, and often ineffective and imprecise, which result in acute and chronic side effects that negatively impact the patient's overall well-being 2. Since Paul Ehrlich promoted the concept of a “magic bullet' in the early 1900s, researchers have attempted to develop targeted platforms that can distinguish between cancer cells and the trillions of healthy cells in the body, and deliver a therapeutic drug dose to cancer cells without harming healthy cells 3. The integration of nanotechnology and targeted drug delivery has opened potential avenues to achieve this goal. With high surface area to volume ratios and unique physiochemical properties, the architecture of nanoparticles allows optimization for the delivery of various imaging and therapeutic agents 4.

**Figure 1:**
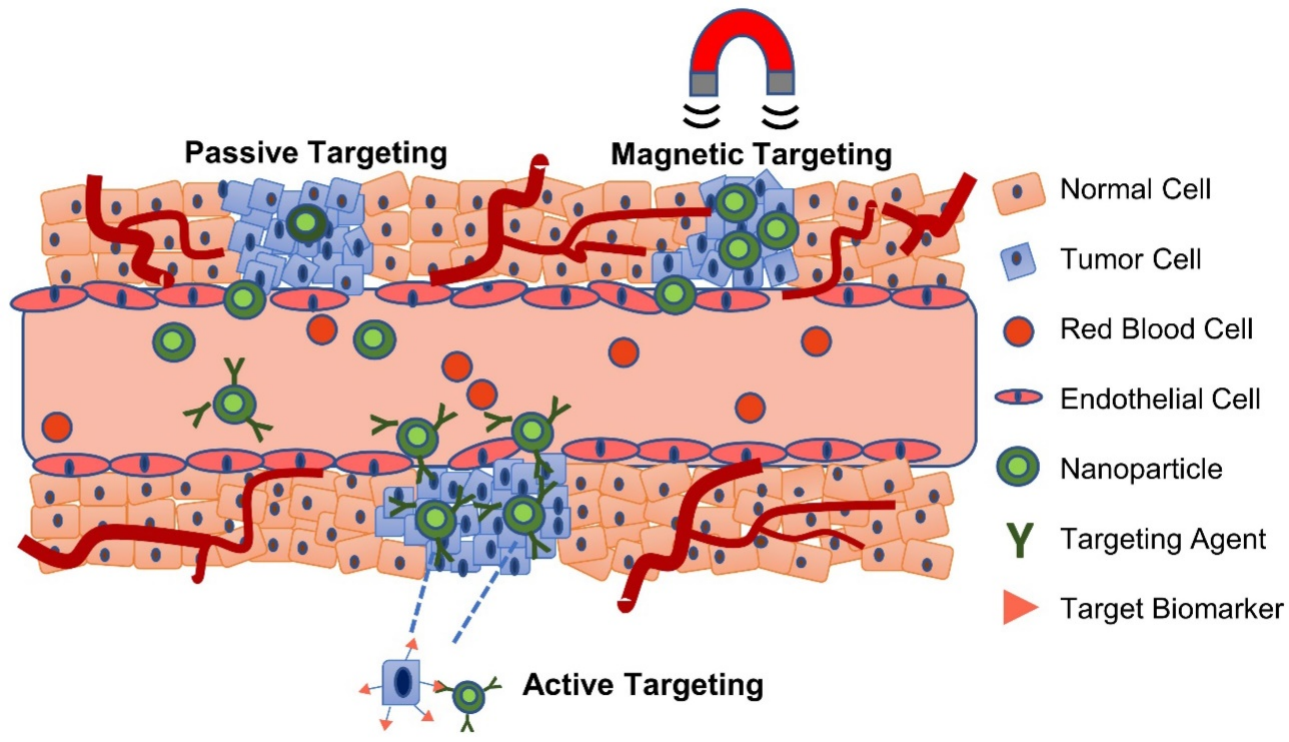
Passive, active and magnetic targeting strategies utilized to enhance the accumulation and efficacy of MRI-traceable, theranostic nanoparticles for targeted cancer treatment. Passive targeting exploits the leaky vasculature and poor lymphatic drainage in the tumor, while active targeting also exploits specific interactions between a targeting agent (e.g. antibody, peptide or aptamer) on the nanoparticle and a nearby biomarker on the target cancer cell. Magnetic targeting, on the other hand, utilizes an externally applied magnetic field to retain magnetic nanoparticles at the tumor site.

Targeting of nanoparticles to tumor sites can be achieved by several general methods: passive, active, or through externally applied forces, as shown in **Figure 1** 5. In passive targeting, nanoparticles selectively accumulate at the tumor site via the enhanced permeability and retention (EPR) effect, a phenomenon utilized by several clinically-approved drug nanoformulations including Doxil^TM^ and Abraxane^TM^ 6. It has been suggested that the EPR effect arises due to a leaky vasculature (having pores of 100 nm to 2 µm in diameter) and poor lymphatic drainage in the tumor - two distinctive characteristics of some neoplastic tissues not present in normal tissues 7. While numerous studies have attributed increased tumor accumulation to the EPR effect, it should be noted that less than 1% of administered nanoparticles accumulate in solid tumors 8, and the vast majority accumulate in other organs - primarily the liver, spleen, and lungs 9. The EPR effect is observed to be highly stochastic; primarily due to tumors having uneven and sometimes absent vascularization, high heterogeneity, a dense stroma, and high interstitial fluid pressure 6. In fact, given the broad variability in the observed EPR effect, there is some controversy regarding its existence 10.

Active targeting utilizes specific interactions between a ligand on the nanoparticle surface and a biomarker, or receptor, on the target cells 11. The extent of these interactions depends on the binding affinity of the ligand to its target, expression level of the receptor on target cells compared to non-target cells, and the rates of internalization and shedding of the receptor following binding 12. Some of the commonly targeted receptors include the epidermal growth factor receptor (EGFR) family which are overexpressed in breast cancer and stimulate different downstream signaling pathways such as angiogenesis, proliferation, and migration of cancer cells 13; transferrin receptor for brain drug delivery 14; and integrin α_v_β_3_, a cell adhesion receptor that is overexpressed in several types of cancer 15. Ligands include antibodies, peptides, aptamers, and other small molecules (*e.g.* folic acid) 16. Antibodies provide high specificity and binding affinity towards the target, but can be difficult to conjugate to nanoparticles, are potentially immunogenic, and are expensive to produce 17. Peptides are small, easy and cheap to produce, and have improved tumor penetration compared to antibodies 18, but suffer from non-specific binding, poor target affinity, and susceptibility to proteolytic cleavage 19. Aptamers are single-stranded RNA or DNA oligonucleotides that bind to a range of targets, including small molecules (*e.g.* DNA or RNA) and tumor surface receptors, with high specificity and affinity 20. In fact, aptamers are considered nucleotide analogs to antibodies but they are cheaper to produce, have less batch-to-batch variability, lower immunogenicity, greater thermal stability, and are easier to modify 21. However, aptamers suffer from rapid degradation by nucleases in the biological environment and their negative charge makes it difficult to select against negatively charged targets 21. Overall, despite their increasing popularity, active targeting strategies suffer from several limitations including patient-to-patient variations in the expression of the target receptor, heterogeneous receptor expression between cancer cells, recognition selectivity, and the likely presence of such receptors on the surface of normal cells which vastly outnumbers cancer cells 6,22.

The use of an external force, such as in magnetic targeting of magnetically-responsive carriers, however, can yield results that are independent of the complex molecular tumor biology 23. Several types of materials, including magnetic nanoparticles (MNPs), magnetic microbubbles, magnetic microcapsules, and magnetic fibers, have been developed for this purpose, and are discussed in detail by Price and colleagues 23. Readers are also directed to work by Zhi and colleagues, which provides an extensive discussion of targeting strategies for superparamagnetic iron oxide nanoparticles (SPIONs) 20.

Theranostic nanoparticles, having integrated therapy and diagnostics in a single platform, have been developed that could potentially provide a more personalized treatment (*e.g.* optimized therapeutic windows, dosages, etc.) by using imaging tools to monitor cancer heterogeneity and adaptability 24. The four primary factors that must be optimized to obtain an efficient, all-in-one theranostic nanostructure are: (a) combined imaging and therapeutic potential; (b) improved therapeutic efficacy versus toxicity; (c) excellent biocompatibility and pharmacokinetic stability; and (d) tumor-specific targeting 25. Imaging modalities that have been explored to track the fate of nanoparticles *in vivo* and obtain structural, functional, and molecular information about the tumor region include magnetic resonance imaging (MRI), computed tomography (CT), fluorescence imaging and positron emission tomography (PET), which differ in detection sensitivity, penetration depths, and image resolution 26. Among these imaging techniques, MRI provides excellent soft-tissue contrast for high spatial resolution imaging of structures deep within the body 27. MRI contrast agents, often administered to enhance the contrast between normal and diseased tissues 28, are divided into two groups: T1-weighted (positive) contrast agents, which produce brighter images by shortening the longitudinal relaxation time of protons; and, T2-weighted (negative) contrast agents, which yield darker MRI images by shortening the protons' transverse relaxation time 29*.*

**Figure 2:**
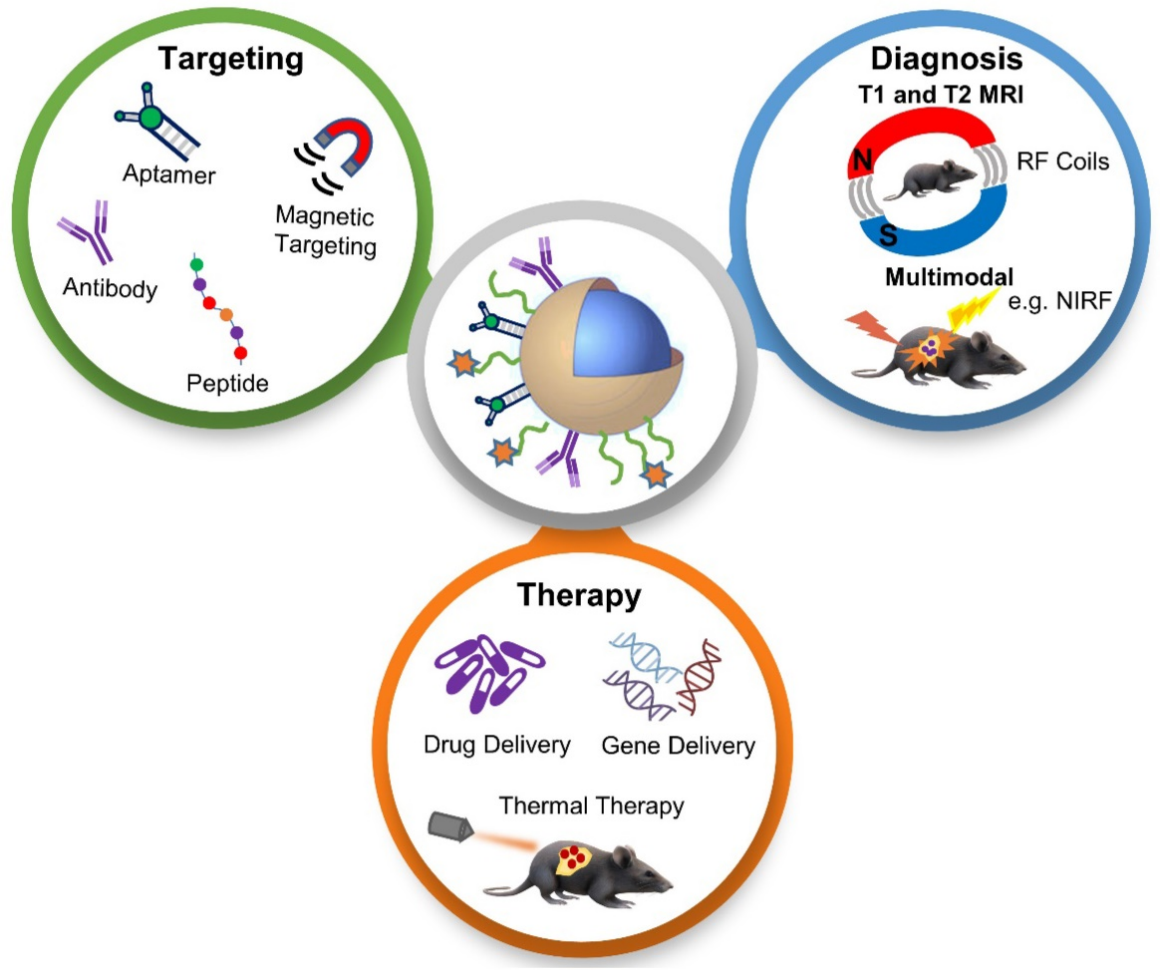
Schematic illustration of the various topics discussed in this review paper, which provides a thorough assessment of recently developed tumor-targeted and in vivo tested nanoparticles for MRI-based diagnosis and anti-cancer therapy, connecting a range of interesting topics including hybrid treatment options, unique MRI-based imaging, and novel targeting strategies. NIRF - near-infrared fluorescence; RF - radiofrequency

In this review paper, we discuss recent developments of “all-in-one” nanotheranostic systems that integrate therapy (*e.g.* chemotherapy, gene therapy, and thermal therapy), MRI-based diagnostics (T1 MRI, T2 MRI, and multimodal imaging), and tumor targeting approaches (active and magnetic targeting), as illustrated in **Figure 2**, with a focus on *in vivo* studies.

## MRI-guided nanoparticles for drug delivery

The era of chemotherapy, *i.e.* the use of cytotoxic agents to ablate rapidly dividing cancer cells, began in the 1940s with the use of Mustine to treat non-Hodgkin's lymphoma 30. Chemotherapeutic agents cause cellular damage through various mechanisms that include incorporating into DNA strands and inhibiting DNA polymerases (*e.g.* Gemcitabine), intercalating within DNA and stopping its replication (*e.g.* Doxorubicin), or forming intra- and inter-strand DNA crosslinks and restricting DNA repair (*e.g.* Cisplatin) 31. These agents, however, suffer from a narrow therapeutic index, high renal clearance, susceptibility to multidrug resistance, and non-specific distribution in the body that results in severe side effects (*e.g.* cardiotoxicity) 32,33. A variety of nanoparticle-based drug delivery systems have been developed to prolong the circulation time of drugs, enhance their efficacy, and reduce side-effects 34. In this section we review recent designs of MRI-guided nanotheranostics and the approaches used to enhance chemotherapeutic efficacy, including: (a) overcoming the various tumor barriers (*e.g.* blood-brain-barrier in glioma) required for deep tumor penetration; (b) overcoming poor active targeting through dual receptor targeting or use of multiple tumor-targeting strategies; (c) enhancing antitumor efficacy through dual-drug delivery; and (d) overcoming limitations of MRI through multimodal imaging. A summary of the most important outcomes is shown in **Table 1**.

**Overcoming barriers:** Several MRI-traceable, tumor-targeted delivery systems have been developed to overcome the complex drug delivery barriers present in different types of cancer and provide real-time assessment of therapeutic responses. For example, the disorganized vasculature and enriched tumor stroma of pancreatic cancers can potentially be overcome by targeting the urokinase plasminogen activator receptor (uPAR) 35. Lee *et al.* developed uPAR-targeted SPIONs for the delivery of the drug Gemcitabine to uPAR-overexpressing pancreatic tumor and stromal cells 36. Controlled release of Gemcitabine from SPIONs was accomplished by enzymatic cleavage of the tetrapeptide (GFLG) linker by overexpressed protease cathepsin B. Systemic administration of the targeted theranostic nanoparticles resulted in significant T2 MRI contrast enhancement and tumor growth inhibition of orthotopic pancreatic tumor xenografts in mice compared to those treated with Gemcitabine only or non-targeted theranostic nanoparticles.

The blood-brain-barrier (BBB), which protects the brain from harmful pathogens, represents another major biological barrier for anticancer treatment. Various methods to bypass or increase permeability across the BBB have been summarized by Israel and colleagues 37. In one study, Hadjipanayis *et al.* explored the potential of Convection Enhanced Delivery (CED), a minimally invasive surgical strategy that generates a pressure gradient at the tip of an infusion catheter placed directly into the brain tumor (*i.e.* bypasses the BBB), to deliver high concentrations of therapeutic agents to focal areas of the brain while reducing systemic toxicity 38. In this study, iron oxide nanoparticles (IONPs) were conjugated to an antibody specific to the epidermal growth factor receptor (EGFR) deletion mutant (EGFRvIII), which is present in malignant gliomas and contributes to tumorigenicity and resistance to chemotherapy. MRI-guided CED of targeted IONPs resulted in an initial spike in the accumulation of the particles within intracranial U87ΔEGFRvIII xenograft tumor as observed by T2-weighted MRI, and continued dispersion seven days after CED. A significant improvement in therapeutic effect and animal survival rate was also observed due to CED-enhanced delivery of the targeted IONPs. In another study, the anti-glioblastoma therapeutic efficacy of poly(lactic-co-glycolic acid) (PLGA)-based nanoparticles loaded with paclitaxel (PTX) and SPIONs was enhanced by the use of magnetic targeting to disrupt the BBB of mice (as observed by MRI images) 39. In this study, magnetic targeting prolonged the median survival rate of orthotopic glioblastoma tumor-bearing mice in comparison to mice in passive targeting and control groups.

Stimuli-responsive nanomaterials that bypass the biological barriers present in the tumor microenvironment have also been developed for targeted intracellular drug delivery. These nanomaterials can be divided into three categories: endogenous (*e.g.* pH- and enzyme) stimuli-responsive, exogenous (*e.g.* light and magnetic field) stimuli-responsive and multi-stimuli responsive nanomaterials 40. In one study, the pH-responsive and tumor-specific peptide H_7_K(R_2_)_2_ was conjugated to the surface of PTX/SPION-loaded liposomes and the pH-responsive, tumor-targeting, and anti-tumor activity properties validated using *in vitro* assays and *in vivo* MRI studies 41. Treatment with the targeted nanoparticles resulted in a significant reduction in mean tumor size (177 ± 85 mm^3^; 90% tumor growth inhibition) at day 31 after implantation compared to mice treated with non-targeted nanoparticles (493 ± 154 mm^3^; 70% tumor growth inhibition). A more thorough discussion on stimuli-responsive cancer theranostic nanocarriers can be found in a recent review by Mi 42.

**Targeting strategies:** Drug delivery systems that target a single cell biomarker are often hindered by a plateau in binding that is reached at a surface density lower than the saturation limit 43. In comparison, nanocarriers with dual-receptor targeting can achieve higher avidity and targeting efficiency by taking advantage of the typical overexpression of several receptors on cancer cells 44. This targeting strategy draws lessons from viruses, which engage in multivalent interactions with multiple membrane receptors before entering host cells 45. Doxorubicin (DOX)-loaded dendrigraft poly-l-lysine (DGL)-MNP modified with RGD- and GX1-peptides, which target the vascular endothelial growth factor and integrin receptors, respectively, demonstrated significantly enhanced HepG2 tumor accumulation of these dual-ligand probes compared to the single-ligand probes 46. In addition, groups treated with these dual-ligand probes demonstrated significantly higher tumor inhibition rate (78.5%) 18 days after treatment compared to groups treated with free DOX (39.6%), MNP-DGL-RGD-GX1 (32.7%), MNP-DGL-RGD-DOX (65.8%), and MNP-DGL-GX1-DOX (68.4%).

Dual active- and magnetic-targeting strategies have also been explored to improve MRI detection and enhance anti-cancer efficacy compared to active or magnetic targeting alone 47. To test this hypothesis, Schleich *et al.* investigated differences in tumor targeting and anti-cancer efficacy of PTX/SPION-loaded PLGA nanoparticles through four different strategies: 1) passive targeting via the EPR effect, 2) active targeting of α_v_β_3_ integrin through RGD grafting, 3) magnetic targeting, and 4) dual active- and magnetic-targeting. The combination of active and magnetic targeting resulted in the highest tumor accumulation (>2.5-fold compared to other groups), the greatest contrast in T2 MRI images, and most potent anti-cancer activity with median survival time of 21.5 days (compared to 10 days with free PTX, 13 days with passive targeting, 14.7 days with active targeting alone, and 15 days with magnetic targeting alone). Similar results were observed by Yang *et al.*, who developed multilayered magneto-vesicles (MuMVs) modified with RGD and DOX and evaluated its efficacy, with and without magnetic targeting, in U87MG tumor-bearing mice 48. Tumors treated with RGD-DOX-MuMVs/magnet+ had the highest T2 contrast and antitumor efficacy due to the synergistic effect of active and magnetic targeting compared to the tumor treated with magnetic or active targeting alone. In another study, magnetic, mesoporous silica nanoparticles carrying a redox-sensitive DOX prodrug and an RGD peptide resulted in enhanced active-targeting, receptor-mediated endocytosis following magnetically-targeted accumulation in α_v_β_3_-integrin positive HeLa tumors 49. This resulted in significantly improved inhibition of tumor growth in mice due to the intracellular activation of the prodrug compared to groups treated with nanoparticles without external magnet and nanoparticles without the RGD peptide and external magnet.

Li and co-workers went one step further by combining magnetic and active targeting with a nuclear targeting mechanism 50. The multistage targeting strategy included: (i) magnetic-guided accumulation of magnetic mesoporous silica nanoparticles in the tumor region; (ii) folic acid (FA)-assisted receptor-mediated endocytosis to the lysosomal compartment of HeLa cells; (iii) surface charge reversal from negative to positive within the acidic lysosome that exposed the nuclear-targeting TAT peptide; which (iv) facilitated the delivery of the DNA-toxic camptothecin-containing nanoparticles into the nucleus of cancer cells.

Functionalizing nanoparticles with cell-derived membranes as a tumor self-targeting strategy has been explored as an alternative means of targeting by reducing nonspecific uptake by the immune system and enhancing tumor delivery 51. Cell membrane coatings derived from numerous cell types, including those from human blood platelets 52 or leukocytes 53, have been used to provide stealth properties to nanoparticles by reducing clearance by the body's immune response and increasing disease accumulation. Cancer cell membranes are ideal for cancer-targeting as they can be collected in bulk (cancer cells are robust and easy to culture), and selectively bind to and are taken up by tumor cells 54. For example, Zhu *et al.* developed a theranostic self-targeting nanoplatform by coating DOX-conjugated IONPs with various types of cracked cancer cell membranes (CCCM) derived from homologous tumors 55. This biomimetic approach relies on the homotypic targeting effect and self-recognition internalization of CCCM-nanoparticles by homologous tumor cells from which the membrane was derived. High accumulation of MNP@DOX@H22 was observed in the H22 tumor of mice bearing two different types of tumors (H22 tumor and UM-SCC-7). In another experiment, antitumor efficacy in UM-SCC-7 tumor-bearing mice was significantly enhanced with MNP@DOX@UM-SCC-7 formulations compared to other cell membrane-coated nanoparticles (MNP@DOX@HeLa and MNP@DOX@COS7). While this approach is promising, challenges for implementation include the need to produce nanoparticles specific for each patient and the development of stringent protocols to ensure high purity of the extracted membrane coatings 54. Readers are directed to a recently published review paper by Harris and colleagues on cancer cell membrane-coated nanoparticles for cancer treatment 54.

**Improving chemotherapeutic efficacy:** Combination chemotherapy, with two or more anti-cancer agents, has been shown to enhance therapeutic efficacy compared to monotherapy 56. Varying pharmacokinetic and biodistribution profiles of drugs, however, results in a suboptimal drug ratio in the tumor 57. For example, it has been shown that 5:1 and 10:1 molar ratios of irinotecan and cisplatin result in optimal antitumor activity against small-cell lung cancer, while molar ratios of 1:2 to 4:1 result in antagonistic effects 58. Nanoparticles can be utilized to maintain the synergistic drug concentrations and molar ratios to achieve optimal therapeutic performance 59. In one study, Kaittanis *et al.* used Ferumoxytol with a prostate-specific membrane antigen (PSMA)-targeting peptide and two drugs (enzalutamide and PI3K inhibitor BEZ235), co-retained within its polymeric coating, for targeted delivery to castration-resistant prostate cancer 60. Intravenous administration of the dual-drug carrying nanoparticles resulted in complete tumor regression of PSMA-expressing, androgen receptor-positive tumor xenografts, in contrast to animals that received an intravenous combination of both drugs in their free form. In another study, Cui *et al.* developed dual active- and magnetically-targeted magnetic PLGA nanoparticles carrying both PTX and curcumin drugs 61. The combination treatment, which synergistically inhibited tumor growth via induction of apoptosis and cell cycle arrest, resulted in significantly enhanced treatment efficacy compared to individual treatment and a combination of free drugs. Efficient brain accumulation was observed in an orthotopic glioma model using MRI studies, synchrotron radiation X-ray studies, and fluorescence imaging.

Another strategy to improve the clinical efficacy of chemotherapy is by targeting transporter-based efflux pumps (*e.g.* P-glycoproteins) responsible for cancer drug resistance 62. Dehvari *et al.* developed stimuli-responsive superparamagnetic iron oxide nanorods (SIONR) encapsulating pluronic F127, a stimuli-responsive block copolymer that inhibits P-glycoproteins and controls the release of PTX in the tumor area, to improve colon cancer therapy 63. Magnetic targeting, which utilizes an externally applied magnetic field to direct magnetic particles into the tumor site, resulted in enhanced accumulation of PTX-F127-SIONR in the tumor region and significantly improved survival time and suppression of CT-26 tumor growth compared to PTX-F127 and docetaxel (DTX).

**Improving diagnostic efficacy through T1 MRI and multimodal imaging:** All nanocarriers discussed so far utilize T2-contrast MRI (dark contrast imaging), which can be difficult to distinguish from other artifact signals caused by pathogenic conditions such as internal bleeding, calcification, metal-deposits or air-tissue boundaries 64. As a result, several studies have focused on developing tumor-targeted T1 (*i.e.* bright) contrast agents. Liu *et al.* developed poly(lactic acid)-based particles with conjugated drug sorafenib, that were targeted to vascular endothelial growth factor receptor-overexpressing cancer and contained gadolinium (Gd) ion chelates for T1 MRI contrast 65. The longitudinal relaxivity of the particles was found to be 3.6 times higher and the MRI imaging time was longer (> 90 min) than that of the commercially available Gd-based contrast agent Magnevist^®^. Additionally, the *in vivo* antitumor effect of the nanomaterial in hepatocellular carcinoma (H22) tumor was markedly higher compared to free sorafenib solution.

Recent concerns of nephrotoxicity and long-term brain deposition in patients exposed to Gd has encouraged researchers to develop safer alternatives to Gd 28. While SPIONs have typically been employed as T2 contrast agents 66, several ultrasmall SPIONs with increased surface area and suppressed magnetization have recently been developed as T1 contrast agents 66-68. In one study, SPIONs and DOX were encapsulated into PLGA-based nanoparticles that served as a tumor-targeted nanotheranostic with T1 contrast properties 69. Nucleolin-targeting AS1411 aptamer (Apt) was further conjugated to the nanoparticles (Apt-NPs) to increase their targeting efficacy. Targeting of nucleolin proteins, which play an important role in cancer cell angiogenesis 70, can block cancer blood vessel growth and deprive tumor cells of vital nutrients. *In vivo* anti-tumor efficacy studies demonstrated significantly enhanced median survival time for C26 tumor-bearing mice treated with Apt-NPs (42 days) versus mice treated with sucrose 10% (21 days), free DOX solution (24 days), and NPs (34 days). In addition, T1-weighted MR images also showed higher accumulation of Apt-NPs in the tumor site in comparison to NPs without aptamers 69. Readers are directed to a review on the development of MNPs as T1 contrast agents written by Bao *et al.* 71

While MRI provides excellent soft-tissue contrast and high image resolution, it suffers from low sensitivity in differentiating healthy and malignant tissues 28. Multimodal imaging has been used to obtain complementary structural/functional/molecular information and draw a more comprehensive diagnostic map describing the complex dynamics of tumor pathogenesis 72. For instance, integration of MRI with imaging modalities that provide higher sensitivity, such as near-infrared fluorescence (NIRF) 73 and PET imaging 74, and the use of multimodal imaging contrast agents significantly reduces data processing time and enhances diagnostic accuracy 75. In one study, Lin and co-workers synthesized dual MRI/NIRF image-guided, cancer-targeted theranostic nanocomposites by decorating the surface of chitosan-coated IONPs (CS-IONPs) with polyethylene glycolated methotrexate (MTX-PEG) and near-infrared (NIR) fluorescent Cy5.5 dye 76. In this case, the anti-cancer drug MTX, which is structurally similar to folate, provided both therapeutic and cancer targeting functionalities to the nanoparticles through its ability to suppress the activity of dihydrofolate reductase enzyme inhibit cell proliferation by interrupting the folate cycle, and ability to target folate receptor-overexpressed tumor cells. *In vivo* NIRF and T2 MRI images demonstrated the enhanced uptake of MTX-PEG-CS-IONPs-Cy5.5 in folate-overexpressing HeLa tumors compared to PEG-CS-IONPs-Cy5.5, while providing improved anti-cancer treatment efficiency with reduced side effects 76. Xu *et al.* developed theranostic multimodal liposomes integrated with SPIONs, quantum dots, and the therapeutic agent cilengitide to target and inhibit overexpressed integrin receptors on C6 glioma cells under magnetic targeting 77. *In vivo* magnetic targeting resulted in enhanced tumor delivery of the agent and effective inhibition of tumor growth, while dual MRI-NIRF imaging allowed for accurate resection of glioma tumors.

In another study, Roy and co-workers developed NIRF/MRI/CT trimodal Fe_3_O_4_ conjugated to the anticancer drug bovine lactoferrin and modified with locked nucleic acid-modified aptamers to provide simultaneous targeting to epithelial cell adhesion molecule (EpCAM) and nucleolin. Oral administration of the targeted formulation improved the survival rate in triple-positive (EpCAM, CD133, CD44) colon cancer xenograft model, with only 10% of mice experiencing tumor recurrence compared to 30% of mice orally fed with non-targeted nanoparticles 78. In addition, all 3 imaging modalities showed maximum contrast in mice treated with targeted formulation versus non-targeted formulation.

Nanoformulations that provide dual T1- and T2-weighted MRI contrast agents have also been developed to enhance MRI sensitivity 75. Wang *et al.* developed magnetic gold nanoparticles (MGs) conjugated with tumor-targeting peptide, LyP-1 (CGNKRTRGC) (MGFs-LyP-1) for targeted trimodality imaging and autophagy-mediated chemosensitization of HepG2 tumor-bearing nude mice 79. Combined T1 and T2 MRI, CT, and NIRF imaging demonstrated enhanced tumor-targeting of MGFs-LyP-1 nanoparticles, which also improved the chemotherapeutic effect of DOX. MGFs-LyP-1 nanoparticles with DOX induced significant autophagy causing HepG2 tumor cell death compared to MGFs-LyP-1 nanoparticles or DOX alone.

## MRI-guided nanoparticles for gene therapy

Gene therapy, which involves the delivery of nucleic acids to modulate the expression of genes involved in tumorigenesis, is a promising cancer treatment modality, especially in conjugation with radiotherapy or chemotherapy 80. Rapid degradation of DNA and RNA by the reticuloendothelial system and limited intracellular delivery, however, limits the efficacy of gene therapy 81,82. Traditional gene therapy also suffers from cytotoxicity, non-specific delivery, and potential immunogenicity 80. The primary objective of nanoparticle design for gene therapy is to improve its circulation time, reduce clearance by the immune system, and selectively deliver therapeutic transgenes to cancer cells while causing minimal harm to normal cells 83. Examples include RGD-coated lipid nanoparticles for tumor-targeted short interfering RNA (siRNA) therapy against glioblastoma 84 and MRI-visible polymeric nanoparticles carrying cell-targeting pullulans for gene delivery into liver cancer cells 85. For an extensive analysis of targeted gene delivery, the readers are directed to a recent review by Subhan *et al.* 86.

Gene therapy can be accomplished by upregulating tumor suppressor genes or gene knockdown using siRNA 87. For example, one study evaluated the potential of a siRNA integrated theranostic system to treat pancreatic ductal adenocarcinoma by targeting polo-like kinases (PLK1) 88. For this purpose, dextran-coated SPIONs were coupled with siPLK1 for silencing PLK1 and halting the cell cycle progression. The nanoparticles were further conjugated with both a tumor-selective peptide targeting the tumor antigen underglycosylated MUC1 and myristoylated polyarginine peptides to mediate endosomal escape of siPLK1 into the cytoplasm. *In vivo* MRI studies demonstrated significant accumulation of siPLK1-carrying SPIONs in the tumor site and efficient PLK1 silencing that significantly inhibited tumor growth. MRI visibility of the formulation allowed for high-resolution spatiotemporal tracking and precise guidance for gene delivery and assessment of treatment efficacy.

Other therapeutic strategies employed include suicide gene therapy, anti-angiogenic gene therapy, genetic immunopotentiation, silencing multi-drug resistance-associated genes (*e.g.* surviving and MDR1), and oncolytic virotherapy 89. The most common suicide gene approach involves the use of herpes simplex virus-thymidine kinase followed by treatment with the antiviral drug ganciclovir that results in failure in DNA replication following the action of thymidine kinase and other enzymes 89. For example, Wang *et al.* synthesized sphere-like (S-M-MSNs) and rod-like magnetic mesoporous silica nanoparticles (R-M-MSNs) loaded with herpes simplex virus thymidine kinase/ganciclovir and compared their performance in MRI-guided, magnetically-targeted and hyperthermia-enhanced suicide gene therapy of hepatocellular carcinoma tumor-bearing mice 90. T2-weighted MRI images demonstrated enhanced gene delivery and hyperthermia performance of R-M-MSNs compared to S-M-MSNs for suicide gene therapy of hepatocellular carcinoma.

In oncolytic virotherapy, viruses are modified to selectively grow and replicate in highly susceptible cancer cells, altering the cancer cell's transcriptional and translational machinery, while leaving normal cells unharmed 89. The result is a combined cancer cell ablation and reactivation of the tumor's immune system 91. The high susceptibility of cancer cells to oncolytic virotherapy is a result of the defective immune responses and abnormal cellular signaling present during tumorigenesis 92. Although virotherapy is FDA approved, systematic delivery of viruses to the tumor microenvironment is still a challenge. To exploit excessive tumor lactate production, Tseng *et al.* developed a lactate-responsive nanocarrier, self-assembled from hyaluronic acid (HA) conjugated with 6-(2 nitroimidazole) hexylamine and loaded with magnetized adeno-associated virus serotype 2 (AAV2) and lactate oxidase (LOX) for controlled release of magnetized AAV2 in the hypoxic, lactate-rich tumor microenvironment 93. Upon reaching the tumor acidic microenvironment, LOX oxidizes lactate molecules, creating a reducing microenvironment within the nanocarrier, converting hydrophobic 2 nitroimidazole to a hydrophilic moiety and electrostatically dissociating the carrier from AAV2. Specific delivery of AAV2 was confirmed with MRI and its enhanced antitumor efficacy validated through the transduction of KillerRed (a photosensitive protein), which enabled significant inhibition in tumor growth *in vivo* through light-triggered virotherapy. A summary of the discussed probes used in gene therapy is shown in **Table 2**.

## MRI-guided nanoparticles for thermal therapy

**Figure 3:**
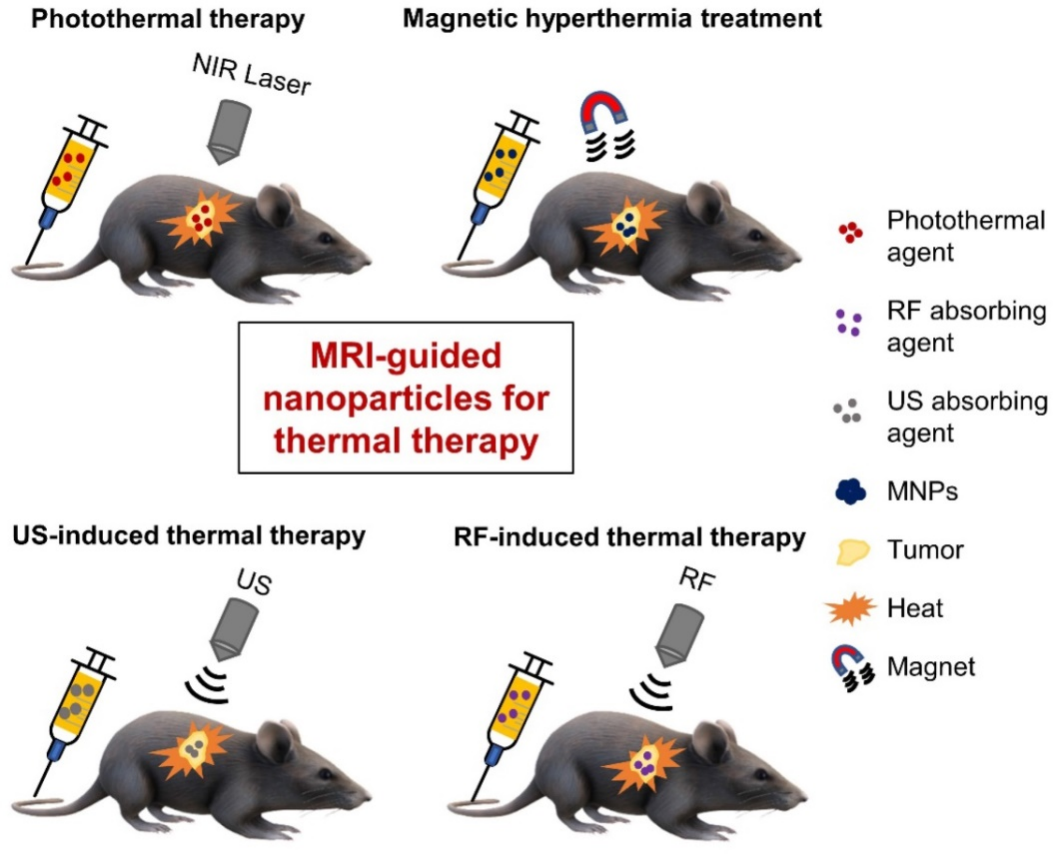
Schematic illustration of various therapeutic modalities used for tumor-targeted thermal ablation of cancer. Several energy sources can be used, including NIR laser for photothermal therapy, alternating magnetic fields for magnetic hyperthermia treatment, US waves for US-induced thermal therapy, and RF energy for RF-induced thermal therapy. MNPs - magnetic nanoparticles; NIR - near-infrared; RF - radiofrequency; US - ultrasound.

Thermal therapy, which involves heating tumors, represents another choice for cancer treatment. The benefits of thermal therapy were first realized in the 19^th^ century, when doctors utilized fever therapies (*e.g.* through the administration of living bacteria to cancer patients) or circulating heated water to shrink tumors 94,95, but poor control and reproducibility often led to failure 96. Interest in thermal therapy has revived over the past few decades after several scientific reports demonstrated significant improvements in cancer therapies, a better understanding of the mechanisms behind temperature-induced cell killing was gained, and new technologies were developed for controlled and localized heating 96. While thermal therapy once referred to whole-body thermal heating, targeted approaches can achieve controlled thermal therapy at the tissue or cellular level. Several energy/heating sources, as shown in **Figure 3**, have been used in thermal therapy and we will examine the use of MRI-guided, tumor-targeted nanoparticles in (i) Photothermal therapy (PTT); (ii) Magnetic hyperthermia treatment (MHT); and (iii) ultrasound (US)- and radiofrequency-induced thermal therapies.

**Photothermal therapy (PTT):** PTT involves the absorption of electromagnetic radiation, typically from a NIR laser, to locally produce thermal energy that induces cancer cell necrosis or apoptosis 97. PTT can be made tissue-specific by targeting of an efficient photo absorbing agent to the tumor region, making targeted PTT a minimally invasive and highly precise treatment modality 98. Since the effectiveness of PTT in cancer therapy is dependent on the incident excitation power (*i.e.* dosage of light delivered) and the concentration and photothermal conversion efficiency of the photothermal agent 99, the ideal photothermal agents possess: (i) excellent optical properties, defined by strong absorption of NIR light and a high absorbance cross-section; (ii) high tumor-homing ability, through passive, active, magnetic or combination targeting; and (iii) minimal toxicity and good biodegradability 99.

Photothermal agents based on organic, noble metal, carbon, or semiconductor materials have all shown good conversion efficiency in the NIR range 100. The readers are directed to reviews by Wei *et al.* 100 and Doughty *et al.* 100 for more extensive coverage of this subject. Yang *et al.* developed a targeted, nanotheranostic agent for synergistic PTT and MRI/fluorescence imaging of cancer by conjugating both HA and CuInS_2_-ZnS quantum dots on the surface of Fe_3_O_4_@prussian blue core-shell nanoparticles 101. Dual targeting with magnetic targeting and CD44 active targeting of HeLa-tumors in mice resulted in highly specific uptake of the nanotheranostic agent at the tumor site, as imaged by MRI and NIRF. The dual-targeting approach resulted in the highest increase in temperature (up to ~49 ᵒC) and *in vivo* PTT efficacy (tumor growth inhibition >90%) against HeLa tumors compared to controls.

Because iron oxide nanoparticles tend to have a low molar absorption coefficient in the NIR range, they are often combined with other photoactive nanomaterials or small molecules for effective PTT 102. In one study, Chen *et al.* developed an upconversion luminescence/MRI-guided photothermal agent consisting of upconversion nanoparticles as the core, a layer of SPIONs as the intermediate shell, and a layer of gold as the outer shell 103. An 8-fold increase in tumor accumulation was observed, by luminescence and MRI, with magnetic targeting compared to without magnetic targeting. In addition, combining NIR laser irradiation with magnetic targeting to treat an *in vivo* breast cancer model resulted in 100% tumor elimination. Magnetic nanomaterials have also been combined with small photothermal agents, such as indocyanine green (ICG) - an FDA approved dye used in fluorescent-guided surgery 104 that is not suitable alone for PTT because of poor photostability, biodistribution, and pharmacokinetics 105. Loading of ICG onto a nanoparticle formulation is known to increase its circulation time and improve delivery to tumors 105. Wu *et al.* developed core-shell magnetite nanoclusters with a coating of polydopamine, and further conjugated PEG and ICG to the surface 106. Application of an external magnetic field resulted in enhanced accumulation of the nanobead in HepG2 liver tumors in mice, as detected by T2-weighted MRI images, and significantly enhanced PTT efficacy due to the combinatory photothermal effect of polydopamine and ICG. Temperature rise was also recorded using an infrared thermal camera; reaching 50.9 °C in tumors treated with magnetically targeted nanobeads compared to 42.3 °C without magnetic targeting.

Various approaches have been explored to further enhance PTT efficacy. Beik *et al.* used MRI-tracing to numerically model the temperature distribution in the tumor region as a pre-treatment tool 107. Other groups developed nanomaterials (*e.g.* Ag_2_S quantum dots and single-walled carbon nanotubes) with peak absorbance in the second NIR (NIR-II) biological window (1000-1350 nm) for enhanced tissue penetration 108. Tsai *et al.* generated core-shell nanoparticles consisting of a double layer of Au/Ag alloy on the surface of IONPs, which exhibited broad NIR absorption that extended to the NIR-II window with a photothermal conversion efficiency of ~28.3% at 1064 nm 109. In vivo results showed that nanoparticles crossed the BBB and magnetic targeting produced sufficient accumulation in U87MG-luc2 brain tumors (detected by T2-weighted MRI images) to reduce cancer cell proliferation when irradiated using a 3 W cm^-2^ 1064 nm diode laser. A digital NIR thermal camera also showed significantly increased temperatures of 45.4 °C in the group treated with the magnetic field versus the group without magnetic targeting (38.3 °C). Photoacoustic imaging (PAI), which detects the ultrasound waves generated by thermoelastic expansion of photothermal agents after absorption of NIR energy, has also been used to monitor temperatures induced by PTT. Several probes have been developed that combine three or more imaging modalities (*e.g.* PAI/MRI plus another imaging modality), including ICG-modified iron nanoparticles for MRI/PAI/fluorescence-guided PTT of MCF-7 xenografted breast tumors 110, and HER-2 targeted DiR-SPIO-PLGA/perfluorocarbon nanoparticles for MRI/US/PAI/NIRF quadrupole imaging and PTT of SKBR3 breast xenograft tumors 111*.* An extensive review of combinations of PTT and PAI is provided by Ge and colleagues 112.

Another strategy to enhance PTT efficacy is through targeting to the cell nucleus. The nucleus, which governs metabolism, reproduction, and the cell cycle, is considered the ultimate and most sensitive organelle for subcellular PTT targeting 113. Peng *et al.* developed a probe consisting of (i) transferrin for active targeting and entry into cancer cells through receptor-mediated endocytosis, (ii) TAT peptide, which binds the importing α and β receptors for nuclear entry, and (iii) fluorescent dyes (fluorescein isothiocyanate or cyanine7) linked to IONPs for MRI/fluorescence-guided PTT 114. Both MRI and fluorescence images indicated a significant accumulation of the probe 8 h post-injection and laser intervention at 12 h post-injection showed significant lung tumor inhibition of about 90% for the dual-targeted probe compared to about 48% and 70% with TAT and transferrin only, respectively.

While standard PTT attempts to achieve a tissue temperature >50 °C, which can also damage normal surrounding tissues 115, several groups have investigated mild photothermal therapy (MPTT) with a temperature rise to ~45 °C. While lowering off-target damage, effective detoxification of the heat stress-induced oxygen radicals by the antioxidative defense system, however, results in low treatment efficacies with MPTT 116. An alternative strategy is to use MPTT as a complementary treatment that sensitizes tumors to immunotherapy and chemotherapy 115,117. The combination of MPTT with chemotherapy, for example, resulted in significantly higher tumor suppression of bone tumor growth and reduction of osteolytic damage at mild temperatures (43 ᵒC) compared to individual therapy following administration of MRI-visible nanoparticles 117. Another strategy used to improve the therapeutic efficacy of MPTT is to target the mitochondria and convert H_2_O_2_ to highly toxic reactive oxygen species (ROS; *e.g.* hydroxyl radicals). Iron ions are known to catalyze the formation of ROS 118 and IONP delivered intracellularly can stimulate the production of ROS 119. In one study, Qiu *et al.* showed that IONPs with mitochondria-targeting iridium(III) complexes (Ir@Fe_3_O_4_ NPs) rapidly damaged cancer cells under photoactivation 120. T2-weighted MRI images of the HeLa tumor-bearing mouse indicated a significant accumulation of Ir@Fe_3_O_4_ NPs at the tumor site and NIR laser irradiation resulted in a mild localized temperature increase to 42 °C and the production of ^•^OH radicals, which efficiently killed cancer cells both *in vitro* and *in vivo*.

**Magnetic hyperthermia treatment (MHT):** MHT utilizes heat generated by MNPs under an alternating magnetic field to selectively kill tumor cells 121. When exposed to an alternating magnetic field, MNPs can generate heat via hysteresis loss (large multi-domain MNPs) or through Neel- and Brownian relaxation losses (typically small, single-core MNPs) 122. The efficiency of MHT depends on the strength and frequency of the magnetic field, solution viscosity, and the size, composition, and concentration of MNPs 123. While MHT has been under development for nearly 6 decades, including technology commercialization with Nanotherm®, it has yet to achieve significant clinical success or routine clinical application 123. A significant shortcoming has been inadequate and uneven heating due to low and heterogeneous concentrations of MNPs within the target tumor. Kozissnik *et al.* estimated that for particles with a specific absorption rate (SAR) of 1,000 W/g and for a 5 mm diameter tumor, a minimum concentration of 650 µg/cm^3^ is required to achieve sufficient hyperthermia 124. The efficacy of MHT can be enhanced by receptor targeting of the MNPs 122. In one study, superparamagnetic gold-nanoparticle clusters were synthesized on a hepatitis B virus capsid and conjugated with an EGFR-targeting peptide 125. The nanoparticles exhibited tumor cell targeting, T2-weighted MRI, and MHT of EGFR‐expressing tumors in mice. In another study, Xie *et al.* developed Mn-Zn ferrite magnetic nanocrystals modified with ICG dye and RGD peptide as a tumor-targeted, multimodal (T2*-weighted MRI/fluorescence imaging) contrast agent for MHT 126. The authors indicated that despite improved targeting with RGD, the particle concentration was still below that needed to induce sufficient heating of tissue and therapeutic MHT was not improved. Future MHT studies may need to consider targeting of both tumor vasculature and tumor cells to enhance accumulation. A more thorough discussion on the use of MNPs for MHT can be found in a recent review by Liu and colleagues 121.

**Ultrasound (US) and radiofrequency (RF)-induced thermal therapy:** Due to the limited tissue-penetrating depth of light, PTT is restricted primarily to the treatment of superficial tumors 127. Ultrasound, on the other hand, provides improved penetration depth and is already used in the clinic for diagnostic (US imaging) and therapeutic (high intensity focused US) applications. In one study, Liu *et al.* fabricated temperature-responsive, anethole dithiolethione-doped magnetic nanoliposomes (AML) loaded with SPIONs and a hydrogen sulfide (H_2_S) pro-drug that transformed into micro-sized H_2_S microbubbles in the tumor tissue upon US irradiation 128. Dual MRI and US imaging confirmed the enhanced tumor uptake of magnetically guided AMLs and their transformation to H_2_S bubbles, in addition to significantly enhanced anticancer efficacy *in vivo*.

MRI-guided focused US (MRgFUS) is a clinically approved procedure that uses anatomical information from MRI scans to localize, control, and target US energy to ablate the target tissue 129. Despite significantly higher penetration depths compared to NIR light, the high US power needed to treat deep lesions and achieve the desired therapeutic efficacy results in severe adverse effects including skin burns and edema. To mitigate the side effects of MRgFUS, Wang *et al.* developed anti-EGFR monoclonal antibody-conjugated SPIONs as a novel approach to improve the MRI sensitivity and ultrasonic energy deposition at low US power 130. T2-weighted MRI showed enhanced accumulation of targeted nanoparticles in EGFR overexpressing H460 lung tumors and MRgFUS tumor-ablative efficacy (monitored through a series of MRI sequences) was enhanced at lower energy levels, leading to fewer side effects. The authors suggested that anti-tumor efficacy was improved due to enhanced heat transfer rate in tumor tissue due to active targeting and alteration of the acoustic tumor microenvironment by SPIONs, which resulted in improved US energy absorption and deposition in the tumor tissues.

Radiofrequency thermal therapy (RTT) is another form of anticancer thermal therapy that relies on the conversion of radiofrequency (RF) energy into heat in the presence of RF absorbing agents. A folate-targeted, thermosensitive liposomal system incorporating Fe_3_O_4_ nanoparticles, fullerene (C60), and DTX was developed for RF-triggered drug release and combined chemotherapy-thermal therapy 131. In this study, the heat generated by functionalized C60 under RF exposure was used not only to eradicate the cancer cells but also to trigger the release of DTX from the liposomes. The multifunctional nanoparticles were able to selectively ablate cancer cells in highly localized regions via combined active and magnetic targeting. A summary of the imaging probes discussed in this section is shown in **Table 3**.

## Reactive oxygen species (ROS)-based cancer therapy

**Photodynamic therapy (PDT):** PDT is a laser-based therapy that utilizes light to excite photosensitizers, which transfer the energy to neighboring oxygen molecules and generates highly toxic ROS 132,133. Similar to PTT, PDT is considered to be less invasive and more targeted compared to chemotherapy and radiotherapy 134, and it has negligible phototoxicity in the dark 132. Many photosensitizers, however, have poor water solubility, are enzymatically degraded, and have poor tumor tissue targeting 135. A variety of nanocarriers, including liposomes, micelles, silica nanoparticles, and SPIONs, have been used to enhance the solubility of photosensitizers in biological media and enhance their treatment efficacy through tumor-targeting strategies. Tang *et al.* developed NIR-light activated upconversion nanoparticles conjugated with chlorine6 and RGD peptide to target αvβ3-integrin overexpressing human U87 glioma tumor 133. In vivo fluorescence imaging and T1-weighted MRI showed significant accumulation of the nanosystem at the tumor site, which effectively suppressed glioma tumor growth. Ding *et al.* developed dextran nanoparticles loaded with Fe_3_O_4_ nanoparticles for NIRF/MRI dual imaging and magnetic targeting, and conjugated with redox-responsive chlorin e6 (Ce6) for PDT treatment of human breast carcinoma (MCF-7) 134. In another study, tumor-targeting polymeric nanoparticles were loaded with Ce6 and Gd^3+^ for PDT therapy of pancreatic tumor xenografts 135. *In vivo* fluorescence imaging and T1-weighted MRI demonstrated significant accumulation of Ce6 and Gd^3+^ at the tumor site and resulted in enhanced PDT efficiency.

**Sonodynamic therapy (SDT):** SDT is similar to PDT but it utilizes US to activate specialized agents known as sonosensitizers to generate ROS to kill cancer cells 136. Due to the higher penetration depths of US compared to light, SDT is more effective in treating deep-seated tumors compared to PDT 137. However, the therapeutic efficacy of SDT can be reduced by several aspects of the tumor microenvironment, such as hypoxia 138. Fu *et al.* integrated ferrate(VI) and protoporphyrin IX into organosilica nanoparticles for sensitizing SDT of hypoxic tumors 138. Studies have also demonstrated the ability of MnO_2_ nanoparticles to alleviate hypoxia by converting tumor-overexpressed hydrogen peroxide into oxygen radicals 139. In one study, Zhu *et al.* developed a tumor-targeted nanosonosensitizer by integrating manganese oxide in hollow mesoporous organosilica nanoparticles loaded with the organic sonosensitizer (protoporphyrin) and decorated with the RGD peptide 127. PAI of mice bearing U87 tumors demonstrated the effectiveness of nanosonosensitizers in suppressing tumor hypoxia, as evidenced by the increase in intratumor oxyhemoglobin saturation from 4.8% to 18.7%. Additionally, T1-weighted MRI showed higher contrast in the tumor volume compared to non-tumor areas, due to the release of paramagnetic Mn^2+^ under the reducing environment, and an efficient 96% tumor inhibition rate in mice after SDT treatment was observed.

**Ferroptosis:** Ferroptosis is a newly discovered form of cell death caused by the accumulation of large amounts of iron in the cell and characterized by the formation of lipid ROS, ultimately causing cell death 140. Ferroptosis has a distinctive morphological, biochemical, and genetic signature from other forms of cell death, including apoptosis, necrosis, and autophagy 141. Morphologically, ferroptosis does not result in the swelling of the cytoplasm or cell/nuclear shrinkage, and instead is characterized by shrinkage of mitochondria, a decreased mitochondrial cristae, and increased bilayer membrane density 142. Biochemically, ferroptosis is characterized by intracellular glutathione depletion, decreased glutathione peroxidase 4 activity, and formation of lipid ROS in a Fenton-like manner, while it is genetically characterized by changes in lipid peroxidation metabolism and iron homeostasis 142. MNPs have been used to accelerate the effect of ferroptosis. In one study, lactoferrin and RGD-conjugated cisplatin-loaded Fe_3_O_4_/Gd_2_O_3_ hybrid nanoparticles were used to accelerate cancer-cell death in orthotopic brain tumors through a ferroptosis-like mechanism 143. The dual ferroptosis-chemotherapeutic effect was aided by dual lactoferrin-receptor-mediated transcytosis and α_v_β_3_-mediated endocytosis, which resulted in significant inhibition of tumor growth, monitored by MRI. In another study, platelet membrane-camouflaged Fe_3_O_4_ nanoparticles were used for combination ferroptosis and immunotherapy to combat tumor metastasis 144. In this case, nanoparticle-mediated ferroptosis was used to sensitize tumors to immunotherapy and repolarize tumor-associated macrophages from an immunosuppressive phenotype to an antitumor phenotype.

## MRI-guided nanoparticles for combination therapy

The heterogeneous and multifactorial nature of cancer requires a multimodal therapeutic approach to overcome drug resistance, reduce the necessary dose, and minimize side effects 145. In this section, we discuss promising nanocarrier-based, MRI-visible approaches for combination therapy summarized in **Table 4** 146.

**Chemotherapy in combination with thermal therapies:** Despite its minimal invasiveness, improved precision, and reduced side effects compared to chemotherapy 147, PTT suffers from non-uniform heat distribution and limited tissue penetration, which often fails to fully eradicate tumors and leads to a higher risk of tumor recurrence 148. PTT has been combined with chemotherapy to achieve synergistic treatment against complex, large, and heterogeneous tumors, with chemotherapeutic drugs providing continuous action to combat cancer recurrence 117. Incorporation of targeting strategies can further enhance the efficacy of combinatorial chemotherapy-PTT, while imaging allows for precise monitoring of therapeutic effect. Wang *et al.* developed Ag-Fe_3_O_4_-based nanoparticles modified with folate for dual MRI/NIRF-guided chemotherapy-PTT that demonstrated an anti-tumor effect greater than that obtained with monotherapies 149. Poly(ε-caprolactone-co-lactide) (PCLA)-PEG-PCLA nanocapsules that encapsulated SPIOs, IR820, and PTX, for magnetically-guided tumor targeting, fluorescence/MRI imaging, and chemotherapy-PTT, showed significant inhibition against 4T1 tumors in mice when combined with magnetic targeting 150. Other examples include EGFR-targeted, Erbitux-conjugated liposomes with manganese-doped IONPs, gold nanorods and DOX for MRI/fluorescence dual-guided chemotherapy-PTT 151; 5-fluorouracil-loaded, PLGA-coated magnetite nanographene oxide for magnetically-guided chemotherapy-PTT suppression of a CT26 colon cancer 152; and magnetically-targeted, polymer hybrid iron oxide nanocapsules containing IR820 and PTX for fluorescence/MRI-guided chemotherapy-PTT 150.

MHT is most effective when used as an adjunct to chemotherapy and radiotherapy, where increased tumor perfusion by the elevated temperatures improves chemotherapeutic delivery and increases oxygen concentrations for enhanced radiotherapy (tumor radioresistance is primarily due to hypoxia) 153. For example, it has been shown that SPION-derived MHT lowers radiation dosage required to kill tumor cells with radiotherapy. In one study, PTX loaded nanoparticles with active targeting ligands showed a significant reduction in tumor volume under magnetophotothermal (combined MHT and PTT) stimulation plus chemotherapy compared to chemotherapy alone, MHT alone, and PTT alone 154. In another study, Li *et al.* developed a magnetically-targeted theranostic nanosystem loaded with DOX for combined MHT and magneto-thermally-responsive drug release to minimize the toxic effects and enhance the targeting efficacy of chemotherapy for liver cancer treatment 155. The nanocarrier displayed temperature-dependent DOX release and significant accumulation at the tumor site under an external magnetic field (as imaged by T2-weighted MRI), which increased uptake of DOX by cancer cells and prolonged retention within the tumor. Additionally, there was a reduced distribution of DOX to the heart and kidneys and enhanced in vivo therapeutic efficacy compared to MHT or chemotherapy alone.

Metastatic triple-negative breast cancer (TNBC), lacking estrogen, progesterone, and Her-2 receptors, is the most aggressive form of metastatic breast cancer and with therapy options limited to chemotherapy, the overall survival remains poor at 13-18 months 156. Due to the different mechanisms of action, there is a rationale to combine targeted chemotherapy with MHT to improve the treatment of metastatic TNBC. In one study, Manigandan *et al.* developed chitosan-based α_v_β_3_-targeted nanomicellar agents comprised of DOX-SPION complexes for combined chemotherapy and MHT against orthotopic TNBC in a mouse tumor model 157. T2-weighted MRI showed enhanced accumulation of targeted nanoparticles at the tumor site compared to non-targeted nanoparticles, and combined MHT and chemotherapy resulted in significant primary tumor regression after 16 days and prevented metastasis.

Targeted chemotherapy has also been used following high-intensity focused ultrasound (HIFU) ablation, which often results in incomplete eradication of deep-seated tumors, and has common side-effects including damage to nearby healthy tissue and skin burn 158. In one study, Tang and co-workers fabricated temperature-responsive theranostic nanoparticles by encapsulating SPIONs, DOX, and perfluorohexane (PFH) into folate-conjugated PLGA NPs for targeted anticancer hybrid therapy 159. HIFU induced a liquid-gas phase transition of PFH to selectively generate PFH microbubbles at the tumor site (as monitored by MRI and US imaging), enhanced the ablation efficacy of HIFU, and provided control release of DOX, which resulted in efficient and synergistic suppression of tumor growth in vivo compared to monotherapies.

**Chemotherapy in combination with PDT:** Feng *et al.* developed magnetic manganese oxide nanospheres for magnetically-guided combined chemotherapy-PDT 160. Chen *et al.* utilized PTX to induce self-assembly of human serum albumin nanoparticles modified with RGD (targeting), Ce6 photosensitizer (PDT and fluorescence tracking), and Mn^2+^ for MRI tracking 161. Dual MRI-fluorescence imaging showed targeting of the particles to tumors and combined PDT-chemotherapy resulted in improved therapeutic efficacy against α_v_β_3_-integrin-positive U87MG tumors compared to the monotherapies. In another example, Feng *et al.* developed DOX-loaded hollow mesoporous copper sulfide (CuS) nanoparticles capped with SPIONs for magnetically targeted, combined PTT-PDT-chemotherapy 162. CuS nanoparticles enable combined PTT-PDT in a single platform by localized surface plasmon resonance and the ability to enhance ROS levels through leakage of copper ions under NIR irradiation. The system, which relied on NIR-responsive controlled DOX release, magnetic targeting, and T2 MRI tracking, resulted in significantly enhanced treatment effects based on the synergistic combination of phototherapy and chemotherapy.

**Figure 4:**
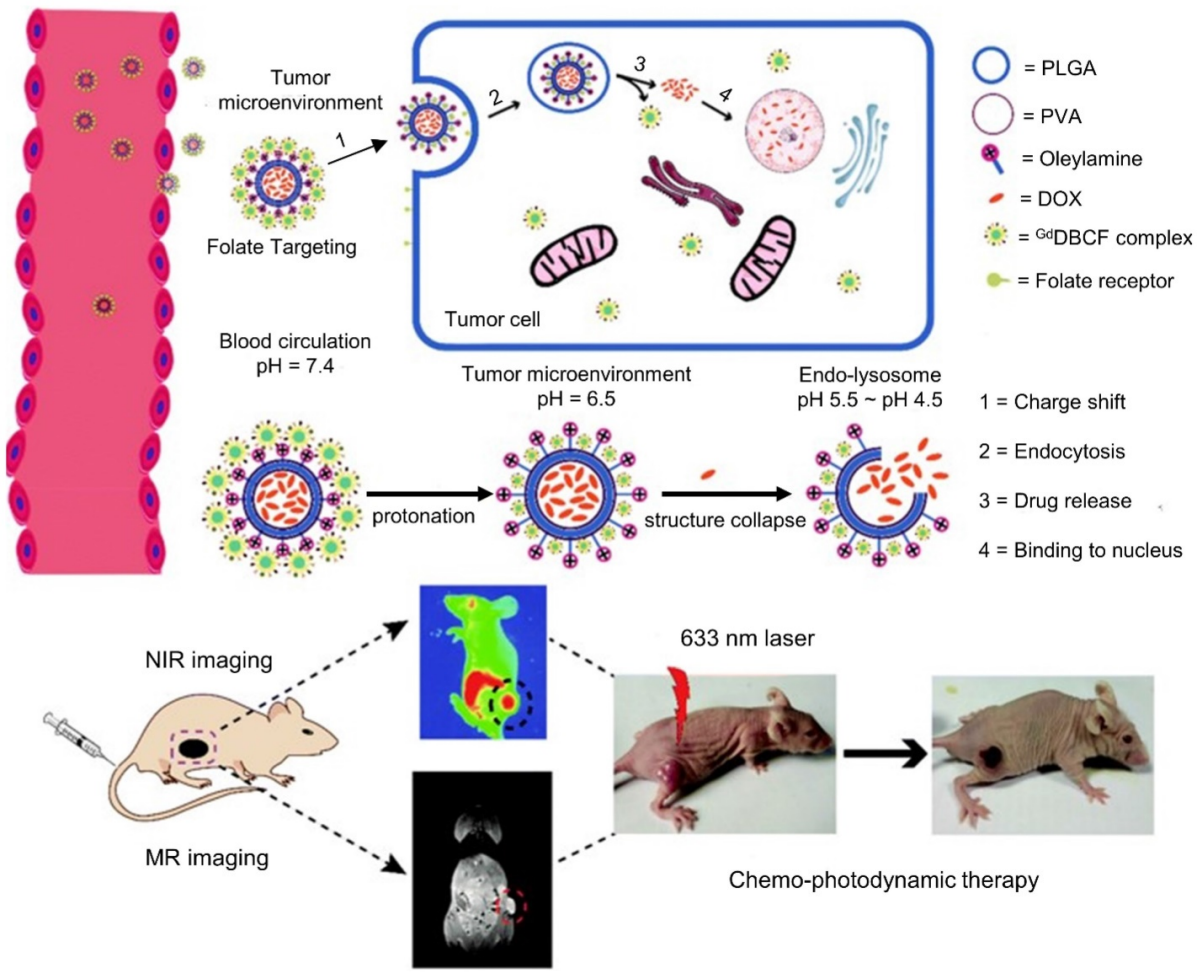
Schematic illustration of charge-switchable nanocapsules displaying multistage pH-responsive behavior for NIRF/MR imaging guided dual chemo-PDT therapy of gastric cancer in vivo. The charge of the nanomaterial changes to positive at mildly acidic condition (pH 6.5) of the tumor microenvironment without releasing DOX or Ce6, and it then releases DOX gradually in the nucleus of tumor cells via the endolysosome (pH 4.5-5.5). Adapted with permission from ref. 25. Copyright 2018 Royal Society of Chemistry (RSC) Publishing.

The variation of pH in the tumor microenvironment (pH 6-7), endosomes (pH 5-6), and lysosomes (pH 4-5) has also been exploited for the delivery of chemotherapy to tumors 25. Strategies include the use of ionizable chemical groups (*e.g.* carboxylic acids and tertiary amines), acid-labile chemical bonds (*e.g.* acetal and acyl hydrazine linkages), anionic and cationic pH-responsive polymers, and pH-sensitive peptides (*e.g.* GALA peptide) 163,164. In one study, Zhou *et al.* developed DOX-encapsulated PLGA, with Ce6 (PDT and fluorescence imaging), Gd-DTPA (T1 MRI tracking) and folate (targeting), as a tumor-targeted, charge-switchable nanomaterial with multistage pH-sensitive behavior for dual chemotherapy-PDT of cancer (**Figure 4**) 25. The charge of the nanomaterial changes to positive at pH 6.5 without releasing DOX or Ce6, and it then releases DOX gradually in the nucleus of MGC-803 cancer cells via the endolysosome (pH 4.5-5.5). This multistage pH-sensitive property resulted in enhanced tumor penetration (as seen in both MRI and NIRF imaging), long retention time, and excellent chemotherapy-PDT synergistic anti-tumor treatment efficacy against MGC-803 tumors in mice, with complete tumor ablation observed after 15 days. Meanwhile, Zhang *et al.* developed black phosphorus quantum dots gated, carbon‐coated Fe_3_O_4_ nanocapsules with pH, NIR, and redox triple-stimuli responsive behavior for continuous DOX release at the tumor site 165. With magnetic- and EGFR-targeting, the nanocapsules efficiently accumulated within A549 tumors and were monitored using both T2-weighted MRI and fluorescence imaging. Furthermore, the nanoprobe significantly improved the therapeutic efficacy of synergistic chemotherapy-PTT compared to chemotherapy or PTT alone, with complete ablation observed after 14 days.

**Combination of PTT and PDT:** Cheng *et al.* developed ultrasmall Gd_2_O_3_ nanoparticles that were labeled with HA (for active tumor targeting), coated with polypyrrole (due to its high photothermal conversion efficiency and PAI property), and conjugated with aluminum phthalocyanine chloride for combined PTT-PDT and fluorescence 64. In vivo studies demonstrated enhanced targeting of the particles to tumors, which significantly enhanced anticancer treatment with combined PTT-PDT therapy compared to any single therapy. In another study, Zhou *et al.* encapsulated ICG, a dual photothermal agent and photosensitizer, in a TiO_2_:Yb-based nanoparticle for trimodal luminescence/MRI/fluorescence imaging and combined PTT-PDT 166. The probe was monitored through upconversion luminescence, MRI and fluorescence imaging, and yielded good antitumor effect in S180 tumor-bearing mice when irradiated with an 808 nm NIR laser.

Targeting subcellular organelles, including the nucleus, mitochondria, and lysosomes, has been shown to significantly enhance phototherapeutic efficacy 167. Zhou *et al.* developed a nucleus-targeting nanoplatform for dual PTT-PDT that resulted in high DNA damage 168. Targeting of the mitochondria has also garnered much attention due to their important role in cell apoptosis and tumorigenesis 169, high abundance throughout the cytoplasm, ease of access compared to the nucleus 170, and hypersensitivity to heat shock and ROS-induced damage 171. For example, mitochondria-targeted PDT is more effective than targeting the cytoplasm as the generated ROS can rapidly cause mitochondrial dysfunction and negatively impact the mitochondria membrane potential - resulting in cellular apoptosis 172. Several mitochondrial targeting strategies have been utilized to improve phototherapies, including the use of lipophilic cations (*e.g.* triphenylphosphonium and cyclometalated Ir(III) complexes), peptides and aptamers (readers are directed to a review on this subject written by Lin *et al.* 173). In one study, Guo *et al.* developed mitochondria-targeted MNPs, which could be tracked by MRI and fluorescence imaging, for dual PTT-PDT 174. The particle consisted of a Fe_3_O_4_ core (for MRI), a polydopamine inner shell (for PTT), and ICG (combined PDT and PTT) encapsulated in mesoporous silica. Additional surface modification with transferrin and triphenylphosphonium, a lipophilic cation, enhanced tumor accumulation (confirmed by dual fluorescence and T2 MR imaging) and further targeting to mitochondria resulted in significantly higher cytotoxicity against an orthotopic lung cancer model.

**Chemotherapy in combination with gene therapy:** Chemotherapy is often hindered by the immunosuppressive tumor microenvironment that impedes drug delivery and results in drug resistance 175,176. Supplementing chemotherapy with gene therapy can alter the genetic makeup of cancers as a means to overcome drug resistance. For example, interactions between Interleukin-4 (IL-4) and its receptor (IL-4R) induce the expression of anti-apoptotic proteins, such as Bcl-xL, which plays a key role in drug resistance and metastatic colonization 177. Guruprasath developed a SPION-based system for dual gene therapy-chemotherapy that carried an IL-4R binding peptide for active targeting and Bcl-xL-targeting siRNA, which sensitized IL-4R-expressing MDA-MB231 breast tumor cells to chemotherapy and enhanced the cytotoxicity of DOX 178. In another study, Qiao *et al.* developed a nanotheranostic system carrying siTGF‐β that down-regulated TGF-β gene expression in glioblastoma and sensitized the tumor to temozolomide-based chemotherapy 176.

As tumorigenesis is associated with abnormal microRNA (miRNA) expression 179, targeting of miRNA, for example, miRNA-21 which contributes to drug resistance, can be used to enhance chemotherapeutic effect 180. Bose *et al.* developed a tumor cell-derived extracellular vesicle (TEV)-based nanosystem, containing gold-iron oxide nanoparticles, for targeted delivery of anti-miRNA-21 to treat 4T1 breast tumors in mice 181. T2-weighted MRI and NIRF images showed significant accumulation of nanoparticles at the tumor site and the co-delivery of anti-miRNA-21 and DOX significantly reduced tumor growth compared to that observed in control groups.

**Combination of immunotherapy with other therapies:** Immunotherapy leverages a patient's immune system to destroy malignant cells 182, for example through targeting of immune checkpoint blockade such as the programmed cell death-1 (PD1) receptor and its ligand PD-L1 183. Nanomedicine has been explored as a means to improve the potency of immunotherapy, which has a ~15% objective response rate across indications, through targeted delivery of immunomodulators and chemotherapies 184-186. Du *et al.* developed an anti-PD-L1 antibody-conjugated liposomal cerasome (*i.e.* ceramic-coated liposome) loaded with PTX for combined immuno-chemotherapy 187. The nanoparticles were further labeled with IRDye800CW and Gd-DTPA to facilitate MRI/NIRF imaging for non-invasive detection of PD-L1 activity. In vivo, T1-weighted MRI and NIRF imaging demonstrated significantly enhanced accumulation of PD-L1 antibody-conjugated nanoparticles in mice bearing 4*T1* and C*T2*6 tumors. In addition, the targeted nanoparticles demonstrated significantly enhanced antitumor activity compared to nontargeted nanoparticles, free PTX, and free PD-L1 antibody. The results suggested that immunotherapy functioned as an adjuvant to improve the efficacy of chemotherapy. In another study, a nanoplatform consisting of SPIONs, anti-PD-1 antibody, perfluoropentane, and GRGDS peptides showed effective ablation of melanoma with combination immunotherapy and PTT 188. Finally, Liu *et al.* designed iron oxide nanorings for combined immunotherapy and MHT to eradicate primary tumors and inhibit metastatic spread 189. Mild MHT activated the host immune system and inhibited the immunosuppressive tumor response through PD-L1 blockage. With the recent approval of anti-PD-L1 antibodies for the treatment of melanoma, methods to quantify PD-L1 expression for treatment planning and to monitor treatment success will be important 190.

## Conclusion

### Summary

Much of the effort to improve the quality of patient care has focused on improving the “targeting” of cancer therapies, by exploiting the molecular signature of cancer cells, to deliver the optimum drug dosage to tumors without harming healthy cells. Nanotechnology offers the opportunity to combine drug targeting with biomedical imaging, specifically MRI with its high spatial resolution, and other treatment modalities to overcome the challenges of cancer diagnosis and therapy. This review paper describes recent developments of multifunctional, cancer-targeted nanotheranostics comprised of targeting molecules, imaging, and therapeutic agents for MRI-based diagnosis and treatment of in vivo tumors. A variety of interdisciplinary topics are covered ranging from novel targeting strategies (*e.g.* dual magnetic-active targeting), combination therapies (*e.g.* combined photo-chemotherapy), and unique imaging opportunities (*e.g.* triple and quadruple multimodal imaging).

### Challenges and outlooks

Despite significant progress in the development of MRI-targeted nanotheranostic platforms and its undeniable potential in predictive, preventive, and personalized medicine, gaps in knowledge continue to hinder translation from bench to bedside and few nanotheranostic systems have undergone clinical trials. This can be due to several factors, including the complexity of the developed hybrid nanosystems; difficulty in predicting their complex effects and interactions with biological systems; species-dependent immune responses and toxicity profiles; difficulty in controlling the pharmacokinetic and biodistribution properties; premature release of the therapeutic cargo in blood and healthy tissue; toxicity concerns; and the significant differences between animal models and human cancer patients 191-193. To improve success rates, recent research has focused on using imaging data to better understand the interactions between nanoparticles and biological systems to optimize tumor targeting and biodistribution.

Other challenges for clinical translation of novel contrast agents is the high dose necessary to achieve the desired diagnostic and therapeutic response, which creates safety and toxicity issues. In addition, the low utilization rate and poor market performance of prior FDA-approved, nanoparticle-based MRI contrast agents, including ferucarbotran and ferumoxides due to the availability of better alternatives 194, has resulted in diminished interest and difficulty in receiving large investments required for the development of new agents. Therefore, a prospective contrast agent should not only have a large market size but must also provide beneficial diagnostic information to justify the high cost associated with its development and use 195. The incorporation of therapeutic capabilities in these agents helps justify the high cost of development as therapeutics have a significantly larger market size compared to pure diagnostic agents. This is especially true when a single agent can provide several functionalities including imaging and combinatorial therapy, such as combining chemotherapy with gene therapy or immunotherapy. An “all in one” nanotheranostic platform, however, requires a complex synthesis route and often shows a premature release of cargo, which can result in severe side effects 193. In the end, clinical translation hinges on proving enhanced efficacy over existing therapies and demonstrating sufficient biocompatibility 196. There is mounting evidence that a combinatorial therapeutic approach is required to combat the heterogeneous and multi-drug resistant nature of aggressive tumors, and that nanoparticles are ideally suited for this purpose 145. While therapies that rely on external sources of energy (*e.g.* thermal therapies and PDT) offer some level of targeting and dose control, they also bring an extra level of complexity that complicates clinical translation and some are limited in their depth of penetration. Proper treatment planning must, therefore, not only consider the strengths and limitations of each modality, but also the physiology, distribution, and type of cancer to be treated.

The majority of targeting strategies discussed in this paper rely on active targeting to “overexpressed” tumor receptors, which requires that the nanoparticles first reach the desired target, mainly by escaping the vasculature through leaky blood vessels, to take advantage of the increased affinity between the carrier and target. In fact, it is generally accepted that, compared to active targeting which can provide a high signal-to-noise ratio for biomarker detection, the permeation of particles through leaky vasculature plays a more dominant role in overall NP accumulation 197. While the EPR effect is typically used to characterize leaky vasculature, it is often tested on rapidly growing xenograft tumors and includes limited experimental data from human patients - complicating clinical translation 6. Recent work by Xu *et al.* showed that actively targeted sub-5 nm IONPs experienced a 6-fold increase in accumulation compared to non-targeted sub-5 nm IONPs, while active targeting of 30-nm IONPs showed a modest 1.15-fold increase in accumulation compared to non-targeted 30 nm 197. Nanoparticle design should also take into consideration the dynamic interactions of nanoparticles with a heterogeneous tumor and the diffusion barriers affecting intratumoral distribution. The design of these actively targeted agents is complex and depends on many factors (*e.g.* nanoparticle's physiochemical properties and architecture, ligand type, and density) 6. While it is true that many targeted receptors/biomarkers tend to be “overexpressed” on the surface of malignant cells compared to normal cells, they are certainly not unique and are often found in nonmalignant tissues as well. This is especially important in human patients where the ratio of the tumor mass to body mass is significantly lower compared to animal models; bringing into question the actual effectiveness of “active targeting” in clinical studies 12. A more effective measure might be to compare the expression of a receptor on a tissue rather than on a cellular level. In addition, it is likely that targeting ligands are only overexpressed on a small percentage of cancer cells, which results in heterogonous accumulation of nanoparticles in the tumor volume and sub-optimal targeting in some regions. Furthermore, while many of the targeting ligands result in cellular internalization, a more extensive evaluation of molecular targets should be based not only on expression but on the rate of internalization and the efficiency of endosomal escape 6. A more thorough understanding of the complex intracellular trafficking mechanisms to optimize the delivery of the therapeutic cargo to the desired destination is necessary to achieve the desired therapeutic effect. Another issue is that many promising receptors for active targeting are identified using cancer cell lines maintained *in vitro*, which may not be representative of what is present in a patient's tumor. A better strategy would be to correlate biomarker expression to tumor grade of human samples. Magnetic targeting is another option, but very few clinical trials have been conducted - mainly due to lack of effective mechanisms for precise delivery of magnetic gradients required to navigate nanoparticles against blood flow (hydrodynamic drag forces in large vessels is significantly greater than magnetic forces) and to deep-seated tumors 23,198. It is entirely possible that all the information needed to achieve optimal targeted therapy is in the literature and that we simply have not yet correlated all the necessary details to solve this complex problem. This task is made more difficult by the large variation between different research groups in nanoparticle systems being evaluated using many different *in vivo* models.

## Figures and Tables

**Table 1:** Summary of tumor-targeted, MRI-traceable nanotheranostics for drug-delivery applications

Targeting strategy	*In vivo* tumor model	Drug	Purpose of investigation	Important outcome(s)	Ref
Integrin-targeting RGD and VEGFR-targeting GX1 peptide	Hepatocellular carcinoma (HepG2)	DOX	Develop a dual integrin & VEGFR-targeted nanotheranostic platform	Groups treated with dual-ligand probes demonstrated significantly higher tumor inhibition rate compared to groups treated with single ligand probes	46
pH-responsive H7K(R2)2 peptide	Breast cancer (MDA-MB-231)	PTX	Develop an MRI-traceable, pH-responsive SPIONs	Tumor size of mice treated with the targeted agent was significantly lower compared to mice treated with non-targeted SPIONs	41
Anti-PSMA peptide targeting PSMA	Prostate cancer (LNCaP)	Enzalutamide and BEZ235	Develop a theranostic agent for targeted dual drug delivery to castration-resistant prostate cancer	Treatment with dual-drug carrying nanoparticles resulted in complete tumor regression of LNCaP tumor xenografts, in contrast to animals treated with a combination of both drugs in their free form	60
MT	Colon cancer (CT-26)	PTX	Develop stimuli-responsive SIONR for controlled release of PTX	Mice treated with PTX- pluronic F127- SIONR showed a higher therapeutic response and higher survival rate compared to mice treated with PTX alone	63
MT	Glioma (U87MG)	PTX	Evaluate MT efficiency and antitumor efficacy of magnetically guided PTX/SPIO-NPs in an orthotopic glioblastoma tumor model	MT resulted in the disruption of the BBB and enhanced accumulation of agent in the brain area of glioblastoma tumor-bearing mice; MT prolonged the median survival rate of glioblastoma tumor-bearing mice compared to PT and control groups	39
RGD targeting αvβ3 integrin & MT	Glioma (U87MG)	DOX	Investigate targeting efficiency differences between PT, AT, MT, and dual AT+MT	Tumors treated with RGD-DOX-MuMVs/magnet+ had the highest T_2_ contrast and highest antitumor efficacy	48
RGD targeting αvβ3 integrin & MT	Cervical cancer (HeLa)	DOX	Develop a targeted theranostic nanoprobe for intracellular redox-sensitive drug release	MT MMSN demonstrated the highest accumulation in the tumor, enhanced MRI contrast and highest therapeutic efficiency	49
T7 peptide targeting transferrin receptor & MT	Glioma cancer (U87-Luc)	PTX and CUR	Develop dual AT+MT NP for codelivery of PTX and CUR to a brain tumor	Combination treatment resulted in significantly enhanced treatment efficacy vs. individual treatment and vs. the combination of free drugs	61
Coating with the source cancer cell membranes	Different types of tumors	DOX	Investigate the self- recognition of CCCM-coated DOX- conjugated Fe_3_O_4_ MNPs by homotypic cancer cells and homologous tumors	Coating the surface of NPs with specific cell membranes obtained from different cancer cell lines led to the self- recognition internalization of NPs by the source cancer cells *in vitro* and homologous tumors *in vivo*	55
AS1411 aptamer targeting nucleolin	Colon cancer (C26)	DOX	Develop anti-nucleolin-targeted magnetic PLGA nanoparticles as a theranostic agent	Median survival time of mice treated with sucrose 10%, free Dox solution, NPs, and Apt-NPs were 21, 5, 34 and 42 days, respectively	69
MT	Glioma (C6)	CGT	Develop theranostic liposome integrated with SPIONs, QDs, and CGT to target and inhibit integrin receptors	MT resulted in enhanced delivery of agent into the tumor region, effective inhibition of tumors, and real-time image-guided accurate surgical resection of glioma tumors	77

Abbreviations: AT: Active targeting; BBB: blood-brain-barrier; CCCM: cracked cancer cell membranes; CGT: cilengitide; CUR: curcumin; MMSN: magnetic mesoporous silica nanoparticle; MT: magnetic targeting; MuMVs: multilayered magneto-vesicles; NP: nanoparticles; PT: passive targeting; PSMA: prostate-specific membrane antigen; PTX: paclitaxel; QDs: quantum dots; RGD: Arg-Gly-Asp; SIONRs: superparamagnetic iron oxide nanorods; SPION: superparamagnetic iron oxide nanoparticles; VEGFR: vascular endothelial growth factor receptor

**Table 2:** Summary of tumor-targeted, MRI-traceable nanotheranostics for cancer thermal therapy

	Imaging probe	Targeting strategy	*in vivo* tumor model	Purpose of investigation	Important outcome(s)	Ref
**PTT**	SPION (T2 MRI)	Mitochondria-targeting lipophilic Ir	Cervical cancer (HeLa)	Develop mitochondria-targeting SPIONs for MRI-guided MPTT	Group of mice treated with 'Ir@Fe_3_O_4_ NPs + NIR' experienced significant tumor growth inhibition	120
	IONP (T2 MRI)	MT	Brain cancer (U87MG-luc2)	Develop a PTT theranostic agent with surface plasmon resonance in the second NIR biological window	MT-enhanced accumulation and NIR irradiation in the second NIR window resulted in a significant reduction of cancer cell proliferation *in vivo*	109
	IONP (T2 MRI) with BQDs (FI)	Combined AT (HA targeting CD44) & MT	Cervical cancer (HeLa)	Develop a probe for dual AT-MT and MRI/fluorescence-guided PTT	Dual AT-MT resulted in highest tumor growth inhibition (>89.95%)	101
	IONP (T2 MRI) with Cyanine7 (FI)	Transferrin targeting TR and nuclear-targeting TAT peptide	Lung cancer (A549)	Dual-targeted MRI/fluorescence-guided PTT with emphasis on nuclear targeting	Treatment with dual-targeted probe resulted in enhanced tumor inhibition rate (90.85%) compared to probe with TAT only (47.91%) and probe with transferrin only (70.6%)	114
	MIL-100(Fe) (T2 MRI) with ICG (FI)	HA targeting CD44	Breast cancer (MCF-7)	Develop a targeted probe for trimodal image-guided PTT	Complete tumor ablation 14 days post-injection of the targeted probe	110
**MHT**	Mn-Zn ferrite MNCs (T2* MRI) with ICG (FI)	RGD peptide targeting αvβ3 integrin	Breast cancer (4T1)	Develop an RGD-targeted agent for MRI/NIRF image-guided MHT	Treatment with targeted NP did not result in a significantly enhanced MHT efficacy vs. treatment with non-targeted NP	126
	SPION (T2 MRI) with Cy5.5 (FI)	Affibody peptide targeting EGFR	Breast cancer (MDA-MB-468)	Develop a targeted nanoprobe for MRI/NIRF image-guided MHT	NP showed excellent targeting, T2 MRI contrast, and MHT of EGFR‐expressing tumors	125
**US-induced thermal therapy**	SPION (T2 MRI)	Monoclonal antibody (Cetuximab) targeting EGFR	Lung cancer (H460)	Develop EGFR-targeted theranostic IONPs to improve the efficacy of MRgFUS	Treatment with the targeted NP enhanced the MRgFUS tumor-ablative efficacy at lower energy levels, leading to fewer side effects	130
**RF thermal therapy**	IONP (T2 MRI)	Combined AT (FA targeting FR) and MT	Melanoma (B16-F10)	Develop a dual AT/MT agent with RF-triggered drug release for chemotherapy-RTT	Selective RF-triggered ablation of cancer cells in highly localized regions via combined AT/MT	131

Abbreviations: AT: Active targeting; BQDs: bovine serum albumin coated cadmium-free CuInS2-ZnS quantum dots; EGFR: Epidermal growth factor receptor; FA: Folic acid; FI: Fluorescence imaging; FR: Folate receptor; HA: Hyaluronic acid; ICG: Indocyanine green; IONP: Iron Oxide Nanoparticles; Ir: iridium(III) cation; MHT: magnetic hyperthermia treatment; MIL-100(Fe): MOF iron(III) trimesate; MNC: Magnetic Nanocrystal; MPTT: mild photothermal therapy; MRgFUS: magnetic resonance-guided focused ultrasound surgery; MRI: Magnetic Resonance Imaging; MT: magnetic targeting; NIRF: Near-infrared fluorescence; NP: nanoparticles; RF: radiofrequency; RGD: Arg-Gly-Asp; RTT: Radiofrequency thermal therapy; SPIONs: Superparamagnetic Iron Oxide Nanoparticles; TR: Transferrin receptor; US: ultrasound

**Table 3:** Summary of tumor-targeted, MRI-traceable nanotheranostics for cancer gene therapy

Imaging probe	Targeting strategy	*In vivo* tumor model	Gene	Purpose of investigation	Important outcome(s)	Ref
SPIONs (T2 MRI)	EPPT1 peptide targeting uMUC1 and MPAPs	Pancreatic cancer (6606PDA)	siPLK1	Evaluate the potential of an siRNA integrated theranostic system targeting PLK1 to treat pancreatic ductal adenocarcinoma	Significant accumulation of siPLK1-StAv-SPIONs in the tumor site; efficient PLK1 silencing that significantly inhibited tumor growth	88
SPIONs (T2 MRI)	MT	Liver cancer (HepG2)	HSV-TK/GCV	Develop an MRI-guided, MT agent for hyperthermia-enhanced suicide gene therapy of HCC	T2-weighted MRI images demonstrated enhanced gene delivery and magnetic hyperthermia performance of rod-like M-MSNs compared to sphere-like M-MSNs for suicide gene therapy	90
SPIONs (T2 MRI)	HA targeting CD44	Lung cancer (NSCLC-H1975)	Virotherapy (magnetized AAV2)	Develop a hypoxia-responsive nanotheranostic agent for tumor virotherapy	Specific delivery of AAV2 to the tumor confirmed with MRI; significantly enhanced tumor inhibition through light-triggered virotherapy	93

Abbreviations: AAV2: adeno-associated virus serotype 2; HA: hyaluronic acid; HCC: hepatocellular carcinoma; HSV-TK/GCV: herpes simplex virus thymidine kinase/ganciclovir; M-MSNs: magnetic mesoporous silica nanoparticles; MPAPs: myristoylated polyarginine peptides; MT: magnetic targeting; NSCLCN: non-small- cell lung cancer; PLK1: polo-like kinases; StAv: streptavidin.

**Table 4:** Summary of tumor-targeted, MRI-traceable nanotheranostics for combination therapy

	Imaging probe	Targeting strategy	*In vivo* tumor model	Purpose of investigation	Important outcome(s)	Ref
**PTT-PDT**	TiO2 (UCL and MRI) with ICG (FI)	HA targeting CD44	Sarcoma (S180)	Develop a trimodal imaging-guided nanoagent for combined PTT-PDT	Probe demonstrated excellent single oxygen yield and photothermal conversion efficacy	166
	Gd2O3 (T1 MRI), Ppy (PAI) and AlPc (FI)	HA targeting CD44	Breast cancer (4T1)	Develop a CD44-targeted, MRI-guided agent for PTT-PDT	Significantly enhanced anticancer treatment with combined PTT-PDT versus any single therapy	64
**Chemo-PTT**	Ferric ion (T1 MRI)	Alendronate targeting Hydroxyapatite	Bone cancer (MDA-MB-231-Luc)	Develop an osteolytic-targeted nanoprobe for chemo-PTT of bone metastasis	Combined therapy resulted in significantly higher suppression of bone tumor growth and reduction of osteolytic damage compared to individual therapy	117
	SPION (T2 MRI) and QD (FI)	anti- EGFR antibody targeting EGFR, MT	Alveolar basal epithelial cells (A549)	Develop a pH, NIR, and redox triple-stimuli responsive, dual AT-MT NP for controlled DOX release & PTT	NP significantly improved antitumor efficacy through combined chemo-PTT (complete ablation after 14 days) compared to monotherapies	165
**Chemo-PDT**	Gd-DTPA (T1 MRI) with chlorin e6 (FI)	FA targeting FR	Gastric cancer (MGC-803)	Develop charge-switchable NP with multistage pH-sensitive behavior for dual chemo-PDT	Multistage pH-sensitive property resulted in enhanced tumor penetration, long retention time and excellent chemo-PDT efficacy	25
**Chemo-PTT-PDT**	SPION (T2 MRI)	MT	NA	Develop MT NP for MRI-guided triple chemo-PTT- PDT	Enhanced PTT and PDT effect and controlled DOX release	162
**Chemo-MHT**	Mn-Zn ferrite MNPs (T2 MRI)	MT	Liver cancer (Huh-7)	Develop MT theranostic NP for combined chemo-MHT	Magnetothermal-responsive Dox release; increased uptake of Dox by cancer cells and prolonged retention;	155
	SPION (T2 MRI) with DOX (FI)	Monoclonal antibody SC-7312 targeting αvβ3 integrin	Breast cancer (4T1)	Develop a nanoagent for combined chemo-MHT and for blocking metastasis of breast cancer cells	Combination therapy resulted in significant primary tumor regression after 16 days, and prevented metastatic migration of cancer into distant organs	157
**Chemo-MHT-PTT**	SPION (T2 MRI) with MHI 148 (FI)	MHI-148 targeting cancer cells	Colorectal carcinoma (CT26)	Develop an image-guided nanoprobe for combined chemo-MHT-PTT	Significant reduction in tumor volume under combined chemo-MHT-PTT compared to monotherapies	154
**Chemo-US induced therapy**	SPION (T2 MRI)	FA targeting FR	Liver cancer (Bel-7402)	Develop targeted and temperature-responsive NP for MRI/US imaging and chemotherapy-HIFU	Combination therapy enhanced the suppression of tumor growth in vivo compared to monotherapies	159
**Chemo-Gene therapy**	SPION (T2 MRI) with Cy5.5 or FITC (FI)	IL4RPep-1 targeting IL-4R	Breast cancer (MDA-MB231)	Develop a dual chemo-gene therapy SPION-based nanodelivery system	The nanodelivery system sensitized IL-4R-expressing MDA-MB231 breast tumor cells to chemotherapy and enhanced the cytotoxicity of DOX	178
	IONP (T2 MRI) with ICG (FI)	TEVs targeting cancer cells	Breast cancer (4T1)	Develop image-guided, targeted NP for anti-miR-21 delivery and enhancement of chemotherapy effect against breast cancer	Combined chemo-gene therapy resulted in significant tumor growth reduction compared to TEV-GION with DOX and DOX alone	181
**Chemo-Immunotherapy**	Gd-DOTA (T1 MRI) with IRDye800CW (FI)	PD-L1 antibody targeting PD-L1	Breast cancer (4T1) and colon cancer (CT26)	Develop targeted, MRI/NIRF traceable NP for combined immuno-chemotherapy	Targeted NP significantly enhanced antitumor efficacy compared to non-targeted nanoparticles, free PTX and free PD-L1 antibody	187

Abbreviations: AlPc: aluminum phthalocyanine chloride; AT: active targeting; DOX: doxorubicin; FA: folic acid; FI: fluorescence imaging; FITC: fluorescein isothiocyanate; FR: Folate receptor; Gd2O3: gadolinium oxide; HA: hyaluronic acid; HIFU: high-intensity focused ultrasound; ICG: Indocyanine green; IL-4R: IL-4 receptor; IL4RPep-1: IL-4R-binding peptide; MHT: magnetic hyperthermia treatment; MNPs: magnetic nanoparticles; MT: magnetic targeting; NIRF: Near-infrared fluorescence; NP: nanoparticles; PAI: photoacoustic imaging; PD-L1: programmed death ligand-1; PDT: photodynamic therapy; Ppy: polymerized conjugated polymer polypyrrole; PTT: photothermal therapy; PTX: paclitaxel; SPION: superparamagnetic iron oxide nanoparticles; TEVs: tumor cell-derived extracellular vesicles; UCL: Upconversion luminescence; US: ultrasound.

## Abbreviations

AAV2: adeno-associated virus serotype 2
CCCM: cracked cancer cell membranes
CT: computed tomography
DiR: DiIC18(7) (1,1'-Dioctadecyl-3,3,3',3'-Tetramethylindotricarbocyanine Iodide
EGFR: epidermal growth factor receptor
EPR: enhanced permeability and retention
FA: folic acid
Gd: gadolinium
HA: hyaluronic acid
ICG: indocyanine green
IONP: Iron oxide nanoparticles
MHT: magnetic hyperthermia treatment
miRNA: microRNA
MNPs: magnetic nanoparticles
MRgFUS: MRI-guided focused ultrasound
MRI: magnetic resonance imaging
MTX: methotrexate
NIR: near-infrared
NIRF: near-infrared fluorescence
PAI: photoacoustic imaging
PDT: photodynamic therapy
PEG: polyethylene glycol
PET: positron emission tomography
PFH: perfluorohexane
PLGA: poly(lactic-co-glycolic acid)
PSMA: prostate-specific membrane antigen
PTT: photothermal therapy
PTX: paclitaxel
SDT: sonodynamic therapy
siRNA: short interfering RNA
SPION: superparamagnetic iron oxide nanoparticles
TNBC: triple-negative breast cancer
US: ultrasound
